# Magnetocaloric Effect of Gd_1-x_Dy_x_ScO_3_ (x = 0, 0.1, 0.2 and 1) Polycrystalline Compounds

**DOI:** 10.3390/ma18122884

**Published:** 2025-06-18

**Authors:** Yuwei Li, Xiukun Hu, Qiong Wu, Yi Zhao, Hangfu Yang, Minxiang Pan, Hongliang Ge

**Affiliations:** 1 Magnetism Key Laboratory of Zhejiang Province, China Jiliang University, Hangzhou 310018, China; lywwyi@163.com (Y.L.); wuqiong@cjlu.edu.cn (Q.W.); minxiangpan@163.com (M.P.); cjlu_hongliang@163.com (H.G.); 2 Hangzhou Quadrant Technology Co., Ltd., Hangzhou 311500, China; y.zhao@quadrant.cn

**Keywords:** magnetocaloric effect, magnetic refrigerant, magnetic properties

## Abstract

This study systematically investigates the magnetic ordering and magnetocaloric properties of a series of polycrystalline compounds, Gd_1-x_Dy_x_ScO_3_ (x = 0, 0.1, 0.2 and 1). X-ray powder diffraction (XRD) analysis confirms that all samples exhibit an orthorhombic perovskite structure with a space group of Pbnm. The zero-field cooling and field cooling magnetization curves demonstrate a transition from antiferromagnetic to paramagnetic phases, with Néel temperatures of about 3 K for GdScO_3_ and 4 K for DyScO_3_. The doping of Dy^3+^ weakened long-range antiferromagnetic order and enhanced short-range magnetic disorder in GdScO_3_, leading to vanished antiferromagnetic transition between 2 and 100 K for the sample of x = 0.2. Using the Arrott–Noakes equation, we constructed Arrott plots to analyze the system’s critical behavior. Both the compounds with x = 0.1 and x = 0.2 conform to the 3D-Heisenberg model. These results indicate the weakened long-range antiferromagnetic order induced by Dy^3+^ doping. Significant maximal magnetic entropy change (−Δ$S_{M}^{Max}$) of 36.03 J/kg K at 3 K for the sample Gd_0.9_Dy_0.1_ScO_3_ is achieved as the magnetic field changes from 0 to 50 kOe, which is higher than that of GdScO_3_ (−Δ$S_{M}^{Max}$ = 34.32 J/kg K) and DyScO_3_ (−Δ$S_{M}^{Max}$ = 15.63 J/kg K). The considerable magnetocaloric effects (MCEs) suggest that these compounds can be used in the development of low-temperature magnetic refrigeration materials.

## 1. Introduction

The magnetocaloric effect (MCE) is an adiabatic temperature change in a magnetic material caused by the variation in magnetization state under the action of an external magnetic field [1]. Since its discovery in the early 20th century, refrigeration technology based on the MCE has attracted extensive attention in the field of science and industry. Compared with conventional gas compression refrigeration technology, magnetic refrigeration technology is not only more energy efficient but also has a lower environmental impact. This advantage makes magnetic refrigeration a promising alternative to traditional refrigeration technologies, especially in applications that require low or very low temperatures, such as medical cryostorage and superconductivity technology. However, to realize the large-scale application and industrialization of magnetic refrigeration technology, the key challenges lie in the selection of magnetic refrigeration materials and the optimization of the MCE.

The ideal magnetic refrigeration material should have a large Δ*S*_M_, a suitable working temperature range, stable mechanical and chemical properties, and low cost and be non-toxic and environmentally friendly. Many types of material have been investigated. Rare earth metal-based materials, e.g., gadolinium and its alloys, are among the earliest research objects in the field of magnetic refrigeration. Gadolinium, in particular, exhibits a significant magnetic entropy change near its Curie temperature (approximately 293 K), making it a classic material for room-temperature magnetic refrigeration [1]. The discovery of the Gd_5_(Si_2_Ge_2_) [2] alloy is considered an important breakthrough in the study of magnetic refrigeration materials. This material enhances magnetic refrigeration performance through the coupling of magnetic and lattice structure changes. The Heusler alloy consisting of transition metals (Ni, Mn, and Co) has become another hot spot in the study of magnetic refrigeration due to its significant magnetic lattice coupling effect [3].

In recent years, rare-earth transition metal perovskite-structured materials (ABO_3_) have shown great application potential due to their unique crystal structures and diverse physical properties. The A- and B-site ions in these materials can be adjusted through chemical doping and element substitution to fine-tune their magnetic and thermodynamic properties, such as Curie temperature and the magnetocaloric effect. Among them, RScO_3_ (where R represents rare-earth elements, such as Gd, Dy, etc.) [4] shows unique magnetic and thermodynamic properties, demonstrating significant potential for magnetic refrigeration applications. For example, GdScO_3_ has an orthorhombic perovskite structure (Pbnm No. 62), and the 4f^7^ electronic configuration of its Gd^3+^ ion gives it not only a high magnetic moment, but also suitable Curie temperature, which makes it excellent in magnetic refrigeration applications. Studies have shown that GdScO_3_ exhibits a significant Δ*S*_M_ up to 39.39 J/kg K near its magnetic transition temperature [5]. In addition to GdScO_3_, DyScO_3_ is also an important perovskite material, showing good magnetocaloric properties and potential applications. It is found that the material exhibits strong magnetic anisotropy and an Ising-type antiferromagnetic structure at low temperatures (3.5 K). Under low magnetic fields (10–20 kOe), significant rotational magnetic entropy changes can be obtained by rotating single crystal, indicating that the DyScO_3_ single crystal is a potential candidate material for low-field magnetic refrigeration in the liquid helium temperature region [6]. Furthermore, our previous studies on rare earth-based chromate showed that GdCrO_3_ and DyCrO_3_ have large MCEs near the phase transition temperature $T_{N}^{R}$ (2.8–3.9 K) [7]. Gd^3+^ and Dy^3+^ ions introduce different critical behaviors, which lead to different dependences of the MCE on the magnetic field. The MCE can also be tuned by co-doping Gd^3+^/Dy^3+^ with chromate compounds [8]. These studies demonstrate the significant potential of co-doping with Dy^3+^ and Gd^3+^ in enhancing magnetocaloric properties. Therefore, it is also worthwhile to systematically study the specific effect of Dy^3+^ doping on the magnetocaloric properties of GdScO_3_ materials.

In this work, the magnetic properties and MCEs of perovskite structure materials GdScO_3_, DyScO_3_, and Dy^3+^-doped GdScO_3_ were investigated. The crystal structure and magnetic phase transition were analyzed. The magnetocaloric effect of these materials was analyzed through isothermal magnetization experiments.

## 2. Experimental Details

A series of polycrystalline compounds, Gd_1-x_Dy_x_ScO_3_ (x = 0, 0.1, 0.2 and 1), were synthesized using a solid-phase sintering method. High-purity (≥99.99%) Gd_2_O_3_, Dy_2_O_3_, and Sc_2_O_3_ powders were weighted according to the nominal stoichiometric ratio, mixed, and thoroughly ground. The mixed powder was pre-sintered at 1000 °C for 24 h and then pressed into pellets with a diameter of 18 mm and a thickness of 2 mm. The pellets were sintered at 1500 °C for 48 h to form good crystal structures. The crystal structures of the samples were characterized by X-ray powder diffraction (XRD, SmartLab SE, Rikkyo Co., Ltd., Toshima, Japan) equipped with a Cu *K*_α_ (λ = 1.514 Å) radiation source at room temperature. The data acquisition range was 10–90° with a scan step size of 0.02°. The surface morphology and elemental distribution of the samples were characterized using Scanning Electron Microscopy (SEM, ZEISS GeminiSEM 360, Zeiss, Germany) and Energy Dispersive Spectroscopy (EDS, OXFORD X-MAX50 Oxford Instruments Technology (Shanghai) Co., Ltd., Shanghai, China), respectively. The magnetic phase transition was investigated by performing zero-field cooling (ZFC) and field cooling (FC) warming magnetization curves under a magnetic field of 100 Oe in the temperature range of 2–300 K using a Physical Property Measurement System (PPMS, Quantum Design, San Diego, CA, USA). Isothermal magnetization curves *M*(*T*, *H*) were recorded in the temperature range of 2–20 K and the magnetic field range of 0–50 kOe for the magnetocaloric effect analysis.

## 3. Results and Discussion

**Figure 1:**
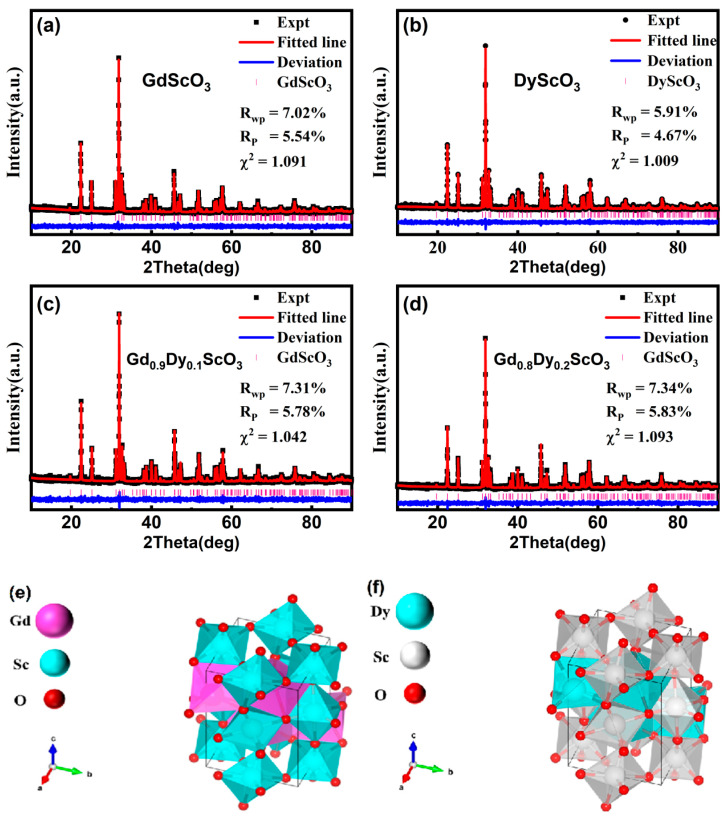
X-ray diffraction patterns for Gd_1-x_Dy_x_ScO_3_ (x = 0, 0.1, 0.2, 1) compounds at room temperature. (**a**) GdScO_3_; (**b**) DyS_C_O_3_; (**c**) Gd_0.9_Dy_0.1_ScO_3_; (**d**) Gd_0.8_Dy_0.2_ScO_3_; (**e**) Representation of the GdScO_3_ crystal structure; (**f**) Representation of the DyScO_3_ crystal structure.

**Figure 2:**
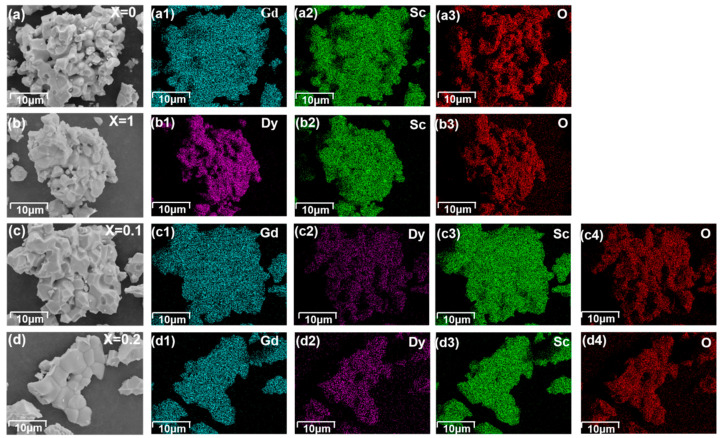
SEM and EDS of Gd_1-x_Dy_x_ScO_3_ (x = 0, 0.1, 0.2, 1). (**a**) GdScO_3_; (**a1**) Gd; (**a2**) Sc; (**a3**) O; (**b**) DyScO_3_; (**b1**) Dy; (**b2**) Sc; (**b3**) O; (**c**) Gd_0.9_Dy_0.1_ScO_3_; (**c1**) Gd; (**c2**) Dy; (**c3**) Sc; (**c4**) O; (**d**) Gd_0.8_Dy_0.2_ScO_3_; (**d1**) Gd; (**d2**) Dy; (**d3**) Sc; (**d4**) O.

**Figure 3:**
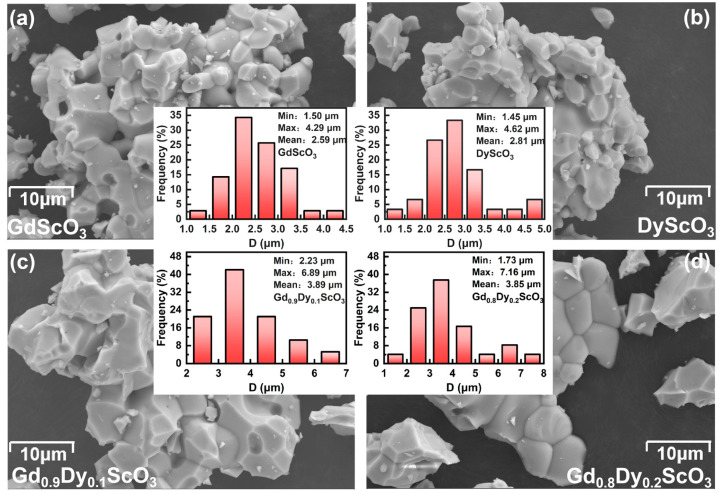
The particle size result graph of Gd_1-x_Dy_x_ScO_3_ (x = 0, 0.1, 0.2, 1). (**a**) GdScO_3_; (**b**) DyS_C_O_3_; (**c**) Gd_0.9_Dy_0.1_ScO_3_; (**d**) Gd_0.8_Dy_0.2_ScO_3_.

Figure 1 shows the XRD patterns of the four samples. Rietveld refinement of the XRD patterns was performed using software (GSAS-II, revision 5758 (svn)) and fitted to the standard diffraction patterns of GdScO_3_ (ICSD #65513) and DyScO_3_ (ICSD #99545), respectively. The results show that all samples exhibit an orthorhombic perovskite structure with a space group of Pbnm. No additional impurity peaks were found in the patterns of Gd_0.8_Dy_0.2_ScO_3_ and Gd_0.9_Dy_0.1_ScO_3_. The main diffraction peaks are highly consistent with the fitting curve, and the baseline is smooth without obvious deviation. The small standard errors R_p_, R_wp_, and χ^2^ shown in Figure 1 indicate the reliability of the refinement results. The lattice parameters were derived from the refinement and listed in Table 1. These parameters are in good agreement with the results reported in the literature [9]. With increasing Dy^3+^ doping, the lattice parameters of the sample decrease, which is in line with the trend of the substitution of small-sized Dy^3+^ ions (0.908 Å) [10] for Gd^3+^ ions (0.938 Å) [11]. The calculation of grain size is ($D=\frac{0.9\lambda }{\beta cos\theta }$) [12], where *D* is the crystallite size, *λ* is the wave length of the X-ray, *β* is the full width at half maximum of the diffraction peak, *θ* is the diffraction angle. The sample shows a polycrystalline structure with an average grain size of 10.58 Å (x = 0), 10.48 Å (x = 0.1), 10.56 Å (x = 0.2), and 17.03 Å (x = 1.0). To further investigate the elemental distributions of Gd_1-x_Dy_x_ScO_3_ (x = 0, 0.1, 0.2, and 1), SEM and EDS were employed to analyze the microstructures and elemental composition of the samples. Figure 2 shows the results obtained using the samples with Gd_1-x_Dy_x_ScO_3_ (x = 0, 0.1, 0.2 and 1). The compounds exhibit relatively homogeneous distributions of Gd and Dy, indicating that Dy doping does not lead to significant phase separation, and a pure phase is formed. Figure 3 presents the particle sizes of the samples: (a) GdScO_3_, (b) DyScO_3_, (c) Gd_0.9_Dy_0.1_ScO_3_, and (d) Gd_0.8_Dy_0.2_ScO_3_. The average particle sizes are approximately 2.59 μm, 2.81 μm, 3.89 μm, and 3.85 μm, respectively.

**Figure 4:**
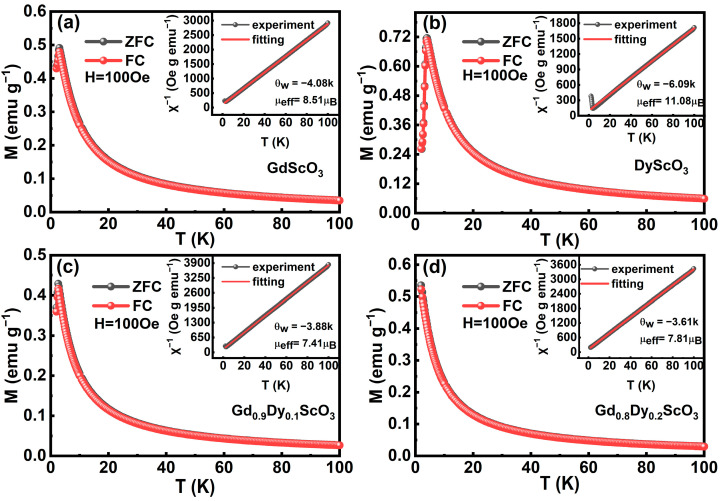
Zero-field cooling curve (ZFC) and field cooling curve (FC) under the field of 100 Oe for Gd_1-x_Dy_x_ScO_3_ (x = 0, 0.1, 0.2, 1) compounds. Insets: temperature dependence of ZFC inverse magnetizations of the Gd_1-x_Dy_x_ScO_3_ (x = 0, 0.1, 0.2, 1) compounds fitted by the Curie-Weiss law under a field of 100 Oe. (**a**) GdScO_3_; (**b**) DyS_C_O_3_; (**c**) Gd_0.9_Dy_0.1_ScO_3_; (**d**) Gd_0.8_Dy_0.2_ScO_3_.

ZFC and FC curves under a 100 Oe magnetic field are shown in Figure 4. For all samples, magnetization gradually increases with the decrease in temperature, showing typical paramagnetic characteristics. When the temperature further decreases, a peak of magnetization was observed for the samples except Gd_0.8_Dy_0.2_ScO_3_. The temperature dependences of reciprocal magnetic susceptibility are plotted in the insets of Figure 4a–d. Above the phase transition temperature, the plots follow the Curie–Weiss law and the Curie–Weiss temperature *θ*_p_ was determined to be −4.08 K, −3.88 K, −3.61 K, and −6.09 K, respectively, indicating antiferromagnetic interaction between rare-earth ions in the samples. The phase transition temperatures (*T*_N_) for the paramagnetic state (PM) to the antiferromagnetic state (AFM) were identified by the presence of hump-shaped anomalies in both ZFC and FC magnetization curves with values of approximately 3 K, 2.8 K and 4 K for GdScO_3_, Gd_0.9_Dy_0.1_ScO_3_, and DyScO_3_, respectively. For the Gd_0.8_Dy_0.2_ScO_3_ sample, no antiferromagnetic transition was observed due to the limitation of the minimum measurement temperature. *T*_N_ of GdScO_3_ reflects the antiferromagnetic interaction between Gd^3+^-Gd^3+^ ions [4]. It is within the range of the phase transition temperatures of previously reported Gd-based compounds, such as GdAlO_3_ (3.87 K) [13] and GdInO_3_ (2.1 K) [14]. *T*_N_ of DyScO_3_ corresponds to the antiferromagnetic interaction between Dy^3+^-Dy^3+^ ions. This is consistent with the phase transition temperature of DyAlO_3_ at 3.92 K [15].

The decrease in *T*_N_ with increasing Dy^3+^ doping content is attributed to the partial destruction of the long-range antiferromagnetic order. The Dy^3+^ ion exhibits strong single-ion anisotropy due to its oblate electron cloud of 4f electrons [16]. The spins lie in the a-b plane and show strong magnetic anisotropy, as reported for the DyScO_3_ single crystal in ref. [6]. Due to the strong magnetic anisotropy of Dy^3+^, the magnetic moment is easily influenced by the local crystal field, making it difficult to antiferromagnetically align with the magnetic moment direction of Gd^3+^. Dy^3+^ ions form localized short-range Dy^3+^-Dy^3+^ and Gd^3+^-Dy^3+^ interactions with surrounding ions and partially destroy the long-range antiferromagnetic coupling of Gd^3+^-Gd^3+^. On the other hand, the local short-range Dy^3+^-Dy^3+^ interaction could increase a certain net magnetic moment in a small external field above the Néel temperatures when the doping amount of Dy^3+^ increases, e.g., x = 0.2 as shown in Figure 4c. The decrease in the absolute value of the Curie–Weiss temperature from 4.08 K (x = 0) to 3.61 K (x = 0.2) also confirms the weakening of the exchange interaction due to the introduction of Dy^3+^.

The effective magnetic moments of GdScO_3_, DyScO_3_, Gd_0.8_Dy_0.2_ScO_3_, and Gd_0.9_Dy_0.1_ScO_3_ were given by [13]

(1) $$\mu_{eff,exp}^{2}=3k_{B}C/N_{A}$$

where *k*_B_, *N*_A_, and *C* are the Boltzmann constant, Avogadro constant, and Curie constant, respectively. The effective magnetic moments of GdScO_3_, DyScO_3_, Gd_0.8_Dy_0.2_ScO_3_, and Gd_0.9_Dy_0.1_ScO_3_ are 8.51 *μ*_B_, 11.08 *μ*_B_, 7.81 *μ*_B_, and 7.41 *μ*_B_, respectively. The theoretical magnetic moments of Gd_1-x_Dy_x_ScO_3_ can be estimated by considering the contribution of Gd^3+^ and Dy^3+^ since Sc^3+^ is a non-magnetic ion. The theoretical magnetic moments of GdScO_3_, DyScO_3_, Gd_0.8_Dy_0.2_ScO_3_, and Gd_0.9_Dy_0.1_ScO_3_ are 7.94 *μ*_B_, 10.68 *μ*_B_, 8.48 *μ*_B_, and 8.21 *μ*_B_, respectively.

The exchange interaction coupling can be estimated by using the Curie–Weiss temperatures:

(2) $$J_{ex}=\frac{3k_{B}\theta_{p}}{2S\left(S+1\right)z}$$

where *S* is the spin quantum number, *θ*_p_ is the Curie–Weiss temperature, and *z* is the nearest neighbor number of each spin. In our calculations, *z* is taken as 6, based on the crystal structure coordination number of Gd (or Dy) ions. The spin quantum number *S* is 7/2 for Gd^3+^ and 5/2 for Dy^3+^, corresponding to their electronic configurations. The calculation gives *J*_ex_ = −0.0449 cm^−1^ for GdScO_3_, which is closer to the reported Gd^3+^-Gd^3+^ interaction in GdAlO_3_ (*J*_ex_ = −0.054 cm^−1^) [13]. The exchange coupling *J*_ex_ is −0.0165 cm^−1^ in DyScO_3_, which is close to the Dy^3+^-Dy^3+^ exchange coupling between DyAlO_3_ compounds reported previously [17]. Additionally, the magnetic dipolar coupling constant *D* using the formula $D=-\frac{\mu_{0}g^{2}{\mu_{B}}^{2}}{4\pi R^{3}}$, where $\mu_{0}$ is the vacuum permeability, *R* is the distance between nearest-neighbor magnetic ions. For GdScO_3_, with *R* = 3.852 Å and *g* = 2, the dipolar coupling constant is approximately −0.031 cm^−1^. For DyScO_3_, with *R* = 3.814 Å and *g* = 4/3, *D* is estimated to be about −0.014 cm^−1^. These values are very close to the calculated exchange couplings *J*_ex_, indicating that the dipolar interaction dominates the effective magnetic coupling in these materials. This further implies that the superexchange interaction between rare-earth ions is quite weak, which explains the low magnetic transition temperatures observed.

**Figure 5:**
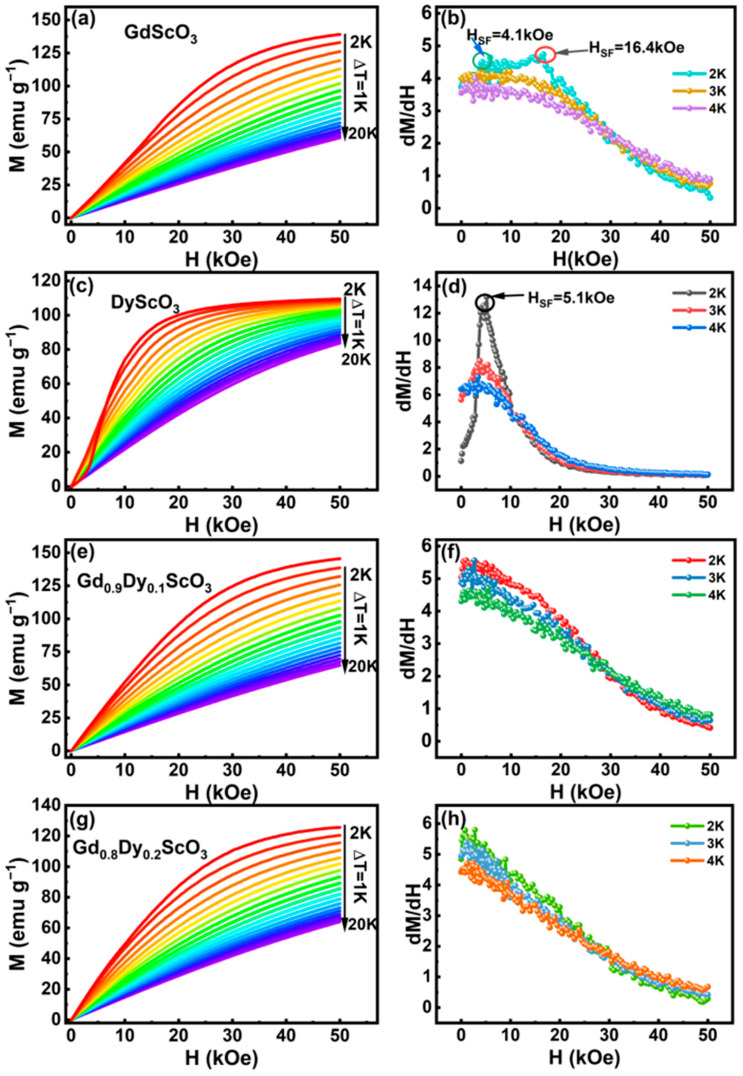
Magnetic field dependences of isothermal magnetizations in the temperature range of 2 to 20 K with a temperature interval of 1 K and the plot of *dM*/*dH*. (**a**) GdScO_3_; (**b**) *dM*/*dH* of GdS_C_O_3_; (**c**) DyScO_3_; (**d**) *dM*/*dH* of DyS_C_O_3_; (**e**) Gd_0.9_Dy_0.1_ScO_3_; (**f**) *dM*/*dH* of Gd_0.9_Dy_0.1_ScO_3_; (**g**) Gd_0.8_Dy_0.2_ScO_3_; (**h**) *dM*/*dH* of Gd_0.8_Dy_0.2_ScO_3_.

The isothermal magnetization curves are shown in Figure 5. For GdScO_3_ (Figure 5a), magnetization increases slowly with an increase in the applied magnetic field, and does not reach saturation under high magnetic fields, which is consistent with the characteristics of antiferromagnetic materials. The magnetization value of GdScO_3_ is 6.223 *μ*_B_/f.u. (139.1 emu/g) at a temperature of 2 K and an applied magnetic field of 50 kOe, which is equivalent to 78.4% of the theoretical saturation magnetic moment of Gd^3+^ ions (7.94 *μ*_B_/f.u.). The existence of antiferromagnetic coupling makes it difficult to completely align the spins with the applied magnetic field even at 50 kOe. To analyze the magnetization process in more detail, the differential of *M* with respect to *H* was performed and the curve is plotted in Figure 5b. There are two clear abnormal points at 4.1 kOe and 16.4 kOe on the curve. The abnormal point at 4.1 kOe is inferred to be the critical field of spin-flop, which is consistent with the previous report on the single crystal GdScO_3_ [18], in which the critical field of spin-flop is about 4 kOe when the magnetic field is parallel to the [010] crystallographic direction. The peak at 16.4 kOe is related to a spin-flop occurring along certain crystallographic directions, which may induce an AFM state to the ferromagnetic (FM) state. Based on the mean field theory, the strength of the exchange field *H*_E_ is deduced to be 30.17 kOe according to $\mu_{B}H_{E}g=6k_{B}T_{N}/\left(S+1\right)$, where *μ*_B_ is the Bohr magneton, *g* is the Landé g-factor, *k*_B_ is the Boltzmann constant, *T*_N_ = 3 K is the Néel temperature, and *S* is the spin quantum number of the magnetic ion. In this work, the values used are *g* = 2 and *S* = 7/2 for the Gd^3+^ ion. The anisotropic field can be calculated using the formula *H*_SF_ = (2*H*_A_*H*_E_)^0.5^ [19], where *H*_SF_ is the value of the spin-flop field. *H*_A_ is 0.27 kOe, similar to that of GdCoO_3_ synthesized using the sol–gel method, where *H*_E_ = 34 kOe and *H*_A_ = 0.33 kOe [19].

The isothermal magnetization curves of Gd_0.9_Dy_0.1_ScO_3_ and Gd_0.8_Dy_0.2_ScO_3_ are shown in Figure 5e,g, respectively. Both have similar magnetization behavior as that of GdScO_3_ but with lower magnetizations of 6.53 *μ*_B_/f.u. (145.521 emu/g) and 5.64 *μ*_B_/f.u. (125.46 emu/g), respectively. As discussed previously, the increase in doping content of Dy^3+^ reduces the content of Gd^3+^, destroys the exchange coupling between Gd^3+^ ions, and forms short-range disorder and a short-range local magnetic state. The formed disordered state makes it impossible to align all magnetic moments completely under a high field, leading to a reduction in the net magnetization, and both abnormal points visible in Figure 5b are no longer visible in Figure 5f,h.

The isothermal magnetization curves (*M*-*H*) of DyScO_3_ at different temperatures are shown in Figure 5c. Ferromagnetic-like magnetization behavior at low temperatures is observed. Dy^3+^-Dy^3+^ weak coupling leads to low stability of the anti-parallel arrangement of Dy^3+^ magnetic moments, making it is easier to disturbed in the external field than GdScO_3_, which is characterized by a rapid increase in magnetization at low fields and saturation at high fields and low temperatures. The derivative curve at 2 K temperature shows a significant peak, appearing at a magnetic field strength of 5.1 kOe, as shown in Figure 5d. This peak corresponds to the occurrence of the spin-flop phenomenon, where the magnetic moment in the antiferromagnet jumps from the initial anti-parallel arrangement to a canted antiferromagnetic arrangement state under the applied magnetic field.

**Figure 6:**
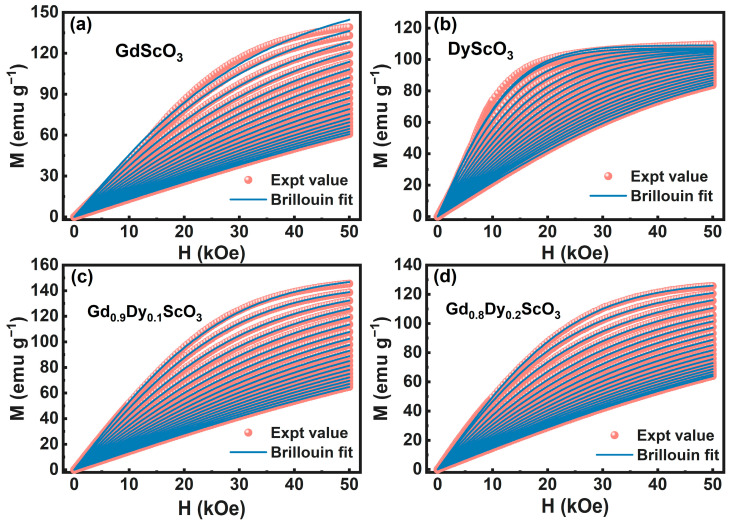
Experimental *M*-*H* data of Gd_1-x_Dy_x_ScO_3_ (x = 0, 0.1, 0.2, 1), in which theoretical fit using Brillouin function were marked out. (**a**) GdScO_3_; (**b**) DyS_C_O_3_; (**c**) Gd_0.9_Dy_0.1_ScO_3_; (**d**) Gd_0.8_Dy_0.2_ScO_3_.

In addition, the Brillouin model is employed to fit the *M*-*H* curve [18]. The formula of Brillouin model fitting is

(3) $$M=Ng\mu_{B}JB_{J}\left(\alpha \right)$$

where $B_{J}\left(\alpha \right)=\frac{2J+1}{2J}coth\left(\frac{2J+1}{2J}\alpha \right)-\frac{1}{2J}coth\left(\frac{\alpha }{2J}\right)$ is the Brillouin function and $\alpha =\frac{g\cdot \mu_{B}\cdot J\cdot H}{k_{B}\cdot T}$. Here, *J* is the total angular momentum quantum number of the magnetic ion. In this work, *J* = 7/2 is used for Gd^3+^ ions. The fitting results are shown in Figure 6. Figure 6a,b show a poor fitting effect in the low-temperature part, while the fitting effect in the high-temperature paramagnetic part is good. Since the Brillouin model is more suitable for fitting the magnetic state above the phase transition temperature, the model is not well suited for describing magnetization behavior with long-range-ordered magnetism and complex local environments. On the contrary, Figure 6c,d show a good overall fitting effect. In the doped samples, due to the high magnetic moment and strong magnetic anisotropy of Dy^3+^, the magnetization behavior is closer to the state of short-range disorder and localized ferromagnetism, and the fitting effect of the Brillouin function model is better.

Critical behavior is typically analyzed by fitting the calculated critical parameters derived from the Arrott–Noakes equation [20]:

(4) $${\left(\frac{H}{M}\right)}^{1/\gamma }=a\left(\frac{T-T_{C}}{T_{C}}\right)+bM^{1/\beta }$$

**Figure 7:**
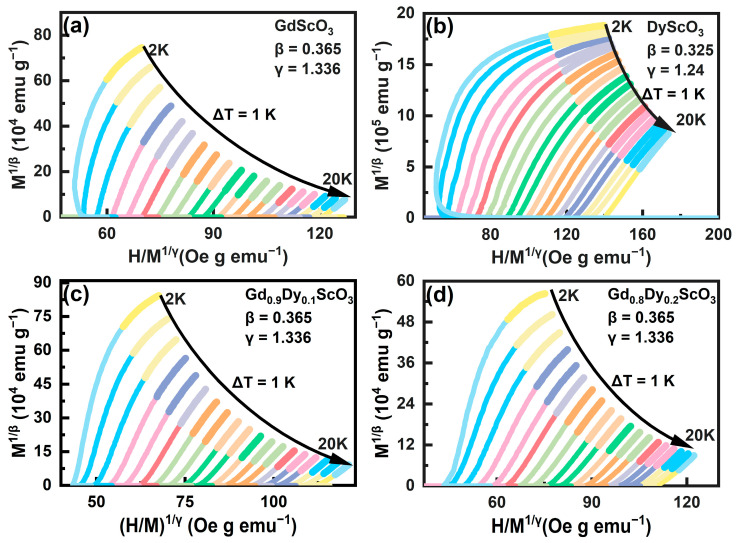
Arrott plots of the Gd_1-x_Dy_x_ScO_3_ (x = 0, 0.1, 0.2, 1) compounds in the temperature range of 2 to 20 K. (**a**) GdScO_3_; (**b**) DyS_C_O_3_; (**c**) Gd_0.9_Dy_0.1_ScO_3_; (**d**) Gd_0.8_Dy_0.2_ScO_3_.

Here, *a* and *b* are treated as constants, while *β* and *γ* represent the critical behavior parameters. In this analysis, we select critical parameters from various theoretical models to construct the Arrott diagram. The conventional theoretical models include the mean field theory model (*β* = 0.5, *γ* = 1), three-phase mean field model (*β* = 0.25, *γ* = 1), 3D-Ising model (*β* = 0.325, *γ* = 1.24), and 3D-Heisenberg model (*β* = 0.365, *γ* = 1.336) [12,21]. The criterion for identifying appropriate critical parameters is linear parallelism at high fields (i.e., the end area of the curves). For the four samples analyzed in this study, the results are presented in Figure 7. Among them, Figure 7a–c show fitting results corresponding to the 3D-Heisenberg model, while Figure 7d present the fitting results corresponding to the 3D-Ising model. However, from the figure it can be seen that the curves at the ends are not completely parallel, indicating that the samples do not fully conform to the proposed theoretical models; therefore, the critical parameters require further refinement. However, due to the lack of data below 2 K, further refinement of critical parameters, as performed in ref. [8], could not be carried out. Nevertheless, the 3D-Heisenberg model reveals the existence of short-range interactions in the system, indicating that the doping of a small amount of Dy^3+^ ions weakened the long-range order and introduced local magnetism.

Magnetocaloric effects were studied based on the isothermal magnetization curves. Magnetic entropy change can be calculated according to Maxwell’s equation [22]:

(5) $$\Delta S_{M}\left(T,H\right)=\int_{0}^{H}{\left(\frac{\partial M}{\partial T}\right)}_{H}dH$$

**Figure 8:**
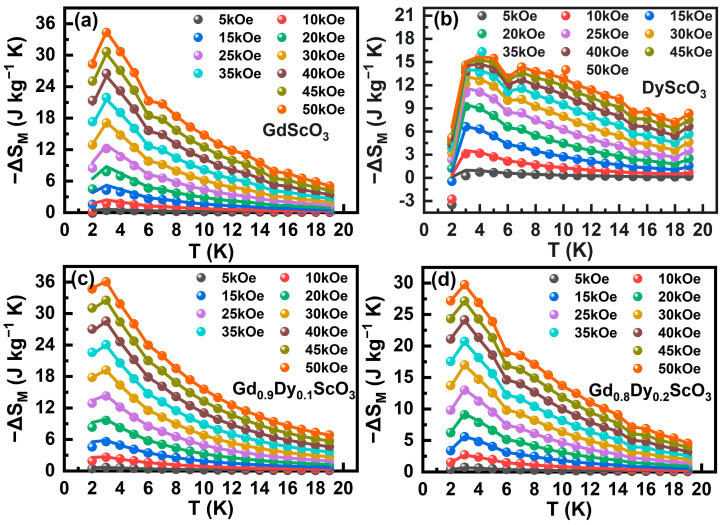
Temperature dependence of magnetic entropy changes −Δ*S*_M_ of the Gd_1-x_Dy_x_ScO_3_ (x = 0, 0.1, 0.2, 1) compounds, the points represent the −Δ$S_{M}^{Max}$ calculated from the measured *M*-*H* curves, while the line represents −Δ$S_{M}^{Max}$ calculated from the *M*-*H* curves fitted using the Brillouin model. (**a**) GdScO_3_; (**b**) DyS_C_O_3_; (**c**) Gd_0.9_Dy_0.1_ScO_3_; (**d**) Gd_0.8_Dy_0.2_ScO_3_.

The specific results are shown in Figure 8. The point is the magnetic entropy change calculated by the actual measurec *M*-*H* results, and the line graph is the magnetic entropy change calculated by the *M*-*H* Brillouin model fitting. Figure 8a shows the magnetic entropy change as function of temperature for the GdScO_3_ sample. The magnetic entropy change increases with the increase in the magnetic field, and both reach the maximum value at the phase transition temperature. Under the action of a magnetic field of 50 kOe, the maximum magnetic entropy change (−Δ$S_{M}^{Max}$) reaches 34.32 J/kg K. Figure 8b shows the −Δ$S_{M\;vs.\;T}^{Max}$ curves of the DyScO_3_ sample. Compared to GdScO_3_, DyScO_3_ shows a wider distribution of magnetic entropy change in the whole temperature range. The maximum magnetic entropy change of DyScO_3_ under the magnetic field of 50 kOe is significantly lower than that of GdScO_3._ This is mainly because the Gd^3+^ ion possesses large spin states (*S* = 7/2), low magnetic anisotropy, and weak spin–spin interactions, which facilitate larger magnetic entropy change [23]. The results are shown in Figure 8c and Figure 8d, respectively. Specifically, the −Δ$S_{M}^{Max}$ of the sample doped with a Dy^3+^ content of 0.1 is 36.03 J/kg K, while the −Δ$S_{M}^{Max}$ of the sample doped with a Dy^3+^ content of 0.2 is 29.74 J/kg K. This result shows that the proportion of disordered states in the low-doped sample is low, and the magnetic moment arrangement of the system still has a certain order, which makes the disturbance more significant under a high field, thus releasing more magnetic entropy. In contrast, as the doping content further increases, the long-range antiferromagnetic network is completely destroyed, the arrangement of magnetic moments is more complex and random, and the disturbance efficiency of the external field to the magnetic moment is significantly reduced, resulting in a decrease in magnetic entropy change. Table 2 lists the phase transition temperatures and −Δ*S*_M_ values for certain Gd- and Dy-based oxides for comparison. There exists a difference in −Δ$S_{M}^{Max}$ between polycrystalline and single-crystal samples. In a sample with the same chemical composition, e.g., DyScO_3_, the MCE of the polycrystalline form is smaller than that of the single-crystal form [6]. This phenomenon is also observed in other materials. On the one hand, some single crystals such as DyScO_3_ [6] and GdVO_4_ [24] show anisotropic MCEs depending on the orientation of the applied magnetic field. In a polycrystalline material with the same chemical composition, the crystal orientations randomly distribute across the sample, and thus, MCEs under a certain magnetic field can be roughly treated as an average result of all crystals; therefore, they are smaller than that of single crystals. It is the case that the MCE of the polycrystalline DyScO_3_ in this work is smaller than that in Ref. [6]. On the other hand, there are larger amounts of grain boundaries and interfaces in polycrystalline than in single-crystal samples, where disorder could exist or magnetic interaction could occur. As reported for GdVO_4_ nanoparticles by M. Ruan et al. [25], a certain amount of surface disorder of nanoparticles could be helpful for increasing MCEs, while an aggregation effect of a small gain size is responsible for the decrease in MCEs. In this work, a small amount Dy doping in GdScO_3_ slightly improved the MCE of the sample, although its −Δ$S_{M}^{Max}$ was lower than that of GdPO_4_ [26], GdVO_4_ [24], and GdFeO_3_ [27]; the compounds studied in this work still exhibit a moderate magnetocaloric effect, showing their potential for low-temperature magnetic refrigeration.

## 4. Conclusions

In summary, we have studied the crystal structure, magnetic properties, and magnetocaloric effect of Gd_1-x_Dy_x_ScO_3_ (x = 0, 0.1, 0.2 and 1). XRD analysis confirmed that all samples preserved the orthorhombic perovskite structure with the space group Pbnm. Dy^3+^ doping did not disrupt the structural integrity of the material, but it influenced magnetic interactions and magnetocaloric behavior. Dy^3+^ doping gradually weakened the long-range antiferromagnetic coupling of Gd^3+^ ions, resulting in the long-range magnetic order of the compound becoming more localized and disordered. This weakening of exchange interactions was reflected in the reduction in the Curie–Weiss temperature and the phase transition temperature as Dy^3+^ doping increased. Critical behavior analysis using the Arrott–Noakes equation revealed that the x = 0 conforms to the mean field model of long-range order, while x = 0.1 and x = 0.2 samples conform to the 3D-Heisenberg, implying short-range magnetic interaction, suggesting that the doping of Dy^3+^ weakened the long-range order. Appropriate variation in local magnetic ordering caused by the small amount of Dy doping is helpful for the improvement of the MCE in GdScO_3_. Magnetocaloric analysis revealed that GdScO_3_ had a −Δ$S_{M}^{Max}$ of 34.32 J/kg K, while Dy^3+^ doping influenced the magnetocaloric properties. Gd_0.9_Dy_0.1_ScO_3_ achieved a higher −Δ $S_{M}^{Max}$ of 36.03 J/kg K, whereas Gd_0.8_Dy_0.2_ScO_3_ showed a reduced value of 29.74 J/kg K.

## Figures and Tables

**Table 1:** Rietveld refinement of lattice parameters data for Gd_1-x_Dy_x_ScO_3_ (x = 0, 0.1, 0.2, 1).

Sample	a (Å)	b (Å)	c (Å)	V (Å3)
GdScO_3_	5.4862	5.7499	7.9345	250.29
DyScO_3_	5.44632	5.72331	7.90631	246.448
Gd_0.9_Dy_0.1_ScO_3_	5.48422	5.74969	7.93414	250.183
Gd_0.8_Dy_0.2_ScO_3_	5.47811	5.74492	7.92845	249.518

**Table 2:** The phase transition temperature (*T*_M_) and maximum value of the magnetic entropy changes (−Δ$S_{M}^{Max}$) of rare-earth based oxides under different magnetic fields. SC and PC represent single crystals and polycrystals, respectively.

Materials	*T*_M_ (K)	H (kOe)	−Δ$S_{M}^{Max}$ (J/kg K)	References
GdScO_3_(PC)	3.04	50	34.32	This Work
DyScO_3_(PC)	4.02	50	15.63	This Work
Gd_0.9_Dy_0.1_ScO_3_(PC)	2.83	50	36.03	This Work
Gd_0.8_Dy_0.2_ScO_3_(PC)	< 2	50	29.74	This Work
DyScO_3_(SC)	3.5	50	21.18	[6]
GdPO_4_(SC)	0.77	70	62.0	[26]
GdMnO_3_(PC)	4.5	50	12	[13]
GdCrO_3_(PC)	2.3	50	30	[28]
GdVO_4_(SC)	3	50	45	[24]
GdVO_4_(PC)	3	50	41.1	[29]
GdAlO_3_(PC)	3.9	50	23	[13]
GdFeO_3_(PC)	3.6	50	38	[27]
Gd_0.8_Dy_0.2_CrO_3_(PC)	3.9	50	31.7	[8]

## Data Availability

The original contributions presented in this study are included in the article. Further inquiries can be directed to the corresponding authors.
